# Correction: Insight into the introduction of domestic cattle and the process of Neolithization to the Spanish region Galicia by genetic evidence

**DOI:** 10.1371/journal.pone.0269578

**Published:** 2022-06-02

**Authors:** Marie Gurke, Amalia Vidal-Gorosquieta, Johanna L. A. Paijmans, Karolina Wȩcek, Axel Barlow, Gloria González-Fortes, Stefanie Hartmann, Aurora Grandal-d’Anglade, Michael Hofreiter

The third author’s name is spelled incorrectly. The correct name is: Johanna L. A. Paijmans. The correct citation is: Gurke M, Vidal-Gorosquieta A, Paijmans JLA, Wȩcek K, Barlow A, González-Fortes G, et al. (2021) Insight into the introduction of domestic cattle and the process of Neolithization to the Spanish region Galicia by genetic evidence. PLoS ONE 16(4): e0249537. https://doi.org/10.1371/journal.pone.0249537
