# Supplementary figures and images for: Insight into the introduction of domestic cattle and the process of Neolithization to the Spanish region Galicia by genetic evidence
Source: PLoS One. 2021 Apr 28;16(4):e0249537. doi: 10.1371/journal.pone.0249537 (PMC8081239; doi:10.1371/journal.pone.0249537)

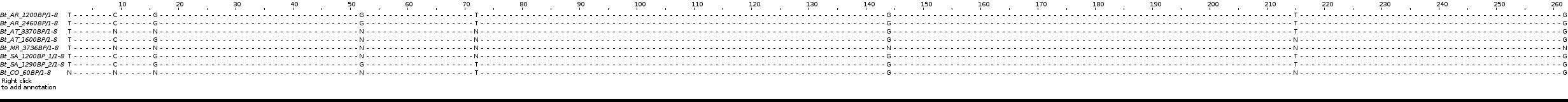

Supplement: S1 Fig — Image shows the the D-loop region of the mitochondrial genomes for all samples aligned. Only the bases in the diagnostic positions are displayed, other bases are displayed as “-”. (PNG) [file pone.0249537.s004.png]
